# Health Workers’ Perceptions and Expectations of the Role of the Pharmacist in Emergency Units: A Qualitative Study in Kupang, Indonesia

**DOI:** 10.3390/pharmacy7010031

**Published:** 2019-03-22

**Authors:** Laila Safitrih, Dyah A. Perwitasari, Nelci Ndoen, Keri L. Dandan

**Affiliations:** 1 Faculty of Pharmacy, University of Ahmad Dahlan, Yogyakarta 55166, Indonesia; lsafitrih18@gmail.com; 2 Pharmacy Unit, Prof. Dr. W.Z. Johannes Hospital, Kupang 85112, Indonesia; nelci.ndoen1975@gmail.com; 3 Faculty of Pharmacy, University of Padjajaran, Bandung 45363, Indonesia; lestarikd@unpad.ac.id

**Keywords:** pharmacy, emergency unit, health workers

## Abstract

**Background**. An essential way to ensure patient safety in the hospital is by applying pharmacy services in emergency units. This strategy was implemented in Indonesia several years ago, with the aim of ensuring that adequate pharmacy services are given to patients in hospitals. To achieve this, pharmacists are required to cooperate with other health workers via inter-professional teamwork. This study intended to identify the perceptions and expectations of health workers with respect to pharmacy services in emergency units. **Methods**. This was a qualitative study, using a phenomenological approach with a semi-structured interview technique to obtain data. This study was performed at the Prof. Dr. W.Z. Johannes Hospital Kupang from June to September 2018. The results of the interviews were thematically analyzed using QSR NVivo software 11. **Results**. The themes identified in this study included: (1) The positive impact of pharmacists in service; (2) Badan Penyelenggara Jaminan Sosial (BPJS) influence; (3) Acceptance of health workers; (4) Medication administration information; and (5) Expectations of health workers. Various perceptions were conveyed by participants regarding the emergency unit services in the hospital’s pharmaceutical department. Data obtained proved that the existence of a pharmacist increased the efficiency of time for services and prevented human error. **Conclusion**. Pharmacists and policy-makers play a significant role in providing appropriate pharmaceutical services in emergency units. Pharmacists also need to improve their quality of practice in accordance with their competence. They must review the patient medical history and physician’s prescriptions, educate the patients and other health workers, so that the workload and service time will be reduced.

## 1. Introduction

One essential way to ensure patient safety in hospitals is by ensuring the presence of pharmacists in emergency rooms. One measure, based on the decision of Accreditation Commission for Hospital (KARS: Komisi Akreditasi Rumah Sakit) in 2012 and 2016 Number 72, aims at ensuring that adequate pharmacy services are given to patients in hospitals. To ensure adequate patient safety in hospitals, pharmacists collaborate with other health workers via inter-professional teamwork. Unfortunately, the relationship between pharmacists and other health personals in developing and developed countries is not cordial [1]. For instance, one study conducted in Canada revealed that a main obstacle to pharmacists in working in hospitals is inadequate support from other health workers [2]. 

Studies about health workers’ perceptions of the role of pharmacists in emergency units have not been conducted in Indonesia. Clinical pharmacist development in Indonesia is still in its infancy. However, problems in emergency units related to medications are increasing, and these can result in medication error. This study aims at identifying the perceptions and expectations of health workers with respect to the emergency pharmacy units in hospitals located in the city of Kupang.

## 2. Methods

### 2.1. Study Design

Qualitative research was undertaken, with a phenomenological approach using a semi-structured interview technique to obtain data. The research was conducted at the Regional General Hospital Prof. Dr. W.Z. Johannes Kupang between June and September 2018. 

### 2.2. Guidelines of the Interview

The questions, which were designed during the study, followed the open-ended question rule. Open-ended questions were purposed to explore the perception of health providers about the pharmacy service in emergency units. The interview guide followed previous studies [3]. However, some questions were designed with the purpose of understanding the impact of pharmacist services on patients’ safety, the cost control, the behavior of health professionals toward pharmacists with respect to high-alert medications, and health professional’s expectations toward pharmacy services in the ward. 

### 2.3. Recruitment Strategy

We used purposive and convenience sampling technique with variations of age, profession, and employment duration. The researcher found information on the participants by contacting a key informant working in the hospital. The researcher directly met the participants at the emergency unit. An informed consent process was conducted by the researcher. Anonymity was used to maintain confidentiality. The interview was conducted directly and recorded based on the permission of the participants. There were two groups of participants: physicians and nurses. Health workers who were willing to take part in the study filled out a form stating their willingness to participate. The researcher ensured that confidentiality was maintained. A face-to-face interview method was conducted using a purposive sampling technique. The interview was held in a closed emergency unit area within the participants’ working hours.

### 2.4. Data Analysis

Data was analyzed using the QSR NVivo 11 software. The examined data were theoretical, which enabled the researcher to determine pre-themes from the research questions. The code was developed from information that was specific or relevant to the research question.

The interview transcript was analyzed by NVivo 11 codebook. The themes were designed based on theoretical thematic analysis, where the codes associated with the research questions were arranged. The themes were arranged based on the Standard of Pharmacy services and the interview. We used open coding without pre-setting and we did not use code from every list; however, we modified the codes during the coding process.

### 2.5. Ethical Consideration

This research received approval from the Research Ethics Commission of the Ahmad Dahlan University (N: 011803044, ED 19.07.2019).

### 2.6. Trustworthiness

This study conducted by researchers with the expertise of lecturers, practitioners, and master’s students. The pharmacy services disclosed in this study were based on the perspectives of physicians and nurses. To minimize study bias, we involved hospital workers to check the data interpretation. An endeavor was made to achieve transferability by explaining the study in more detail. Confirmability was conducted by reflexivity, involving an explanation of the motive, background, perspective, and study finding implications. The themes were designed by LS and reviewed by DA and one external reviewer. Data credibility was guaranteed using member check with transferability used to give a detailed description [4].

## 3. Results

We present our study results according to themes, using narratives and tables with examples of direct quotes from the participants. The narratives of the quotes from physicians and nurses are presented according to the themes. Some narratives are separated with respect to physicians and nurses because of the different perspectives between them, especially in terms of the theme of acceptability of the pharmacist’s role among other health workers. We encoded the physician as “P” and the nurse as “N”.

**Participant:**

A total of eight participants consisting of medical practitioners and nurses participated in the research. The participants’ characteristics are shown in Table 1. The themes identified in this study are presented in Table 2. 

### 3.1. Positive Impact of Pharmacists on Services

#### 3.1.1. Time Efficiency

The presence of pharmacists in emergency units has a positive impact on patients and health workers and it is an essential contextual factor for health workers. The efficient of pharmacy services can improve the time efficiency. With the presence of the pharmacist, faster service is provided to patients.

For example: a patient with heart failure, will need furosemide to lower their breathing rate. But what will happen a situation where there is no pharmacist to prescribe the drug, will the patient be left to die?-P1

We have to wait for a moment, when we have to get the medicine from the pharmacy—N3.

#### 3.1.2. Medication Error Prevention

Installing an emergency medical system is essential in order to prevent human errors associated with medication. Pharmacist has competency in reviewing the prescription, including to recalculate the dosage based on the patients’ condition. This competency is expected by the nurse to be applied in the Emergency unit.

Giving the wrong prescription, can endanger the lives of patients, and the effect can be very fatal—N2

Participants realized that they could make a medication error both in the prescription and administration phases. Participants opined that the presence of pharmacists in the emergency department influences patient’s safety as they tend to review and re-check a prescription to ensure the drugs received are safe and appropriate for consumption.

In my opinion, pharmacists have a huge influence on drug prescription, because drugs need to be properly checked and given with the right prescription—P2

Patient safety at the emergency department is crucial. Human error can occur and it is possible for the pharmacist to make mistakes. Therefore, it is important to crosscheck all prescriptions given to avoid human error.

I tried digging it again, by asking him if he was sure the prescription was ok—P3

We are already accustomed to double checking our prescriptions. Whenever I give out prescription it is cross checked by the pharmacist and if ok, given to the nurse, who also checks and issues according to instructions. Hence, it must be safe—P4

### 3.2. Effect of National Health Insurance

#### 3.2.1. Drug Supply Limitation

In this research the participants expressed the influence of the health financing system on medicine management and the availability of medication in hospitals. There tend to be many issues related to the unavailability of drugs, especially those guaranteed by insurance. Participants stated that one of the administrative barriers which was related to pharmacist work was insurance. When the patients visit the hospital, the pharmacist checks the insurance of the patients as a guarantee. After that, the medicine is delivered to the patients. According to the participants, this situation can hold up service. Thus, insurance is a barrier to services.

Usually the BPJS drugs sometimes run out in government hospitals. That’s the problem, so we ask them first—P1

Sometimes, when the drugs are not available, we have to buy the drugs outside of the hospital—N2

#### 3.2.2. Dilemma

BPJS can influence the service system in the emergency unit. Before delivering the medicine to the patients, the pharmacist should make sure that the patients is under the guarantee of BPJS. This situation should not become the obstacle in the delivery of medicine.

Actually it doesn’t violate the standard operating procedure (SOP) defined by BPJS, however, in the most of the patient’s times, they opt for payment immediately they step into a medical facility. So these conditions has been shown to prevent such actions—P3

In contrast to this perception, this system is more of a dilemma for other participants where they cannot sue the pharmacist. This is because most times after the drug is given, the guarantee requested is not available and the patient fails to pay. This can be detrimental to the hospital. According to one respondent, services can be more flexible for patients with insurance because health workers do not need to think about their patient’s medication costs.

With respect to costs, one of the roles of pharmacists is to ensure that patients make full payment at the hospital; however, this research reveals the existence of a BPJS (national insurance) system, in which participants fail to notice any form of reduction in health costs owing to the presence of a pharmacist in the emergency unit.

In terms of costs, the pharmacist controls the hospital, but this research revealed that with the existence of a BPJS system, participants failed to witness a reduction in health costs. By using BPJS, patients paid nothing, so the reduction of the costs due to the pharmacist’ role was not clear.

If it’s here because the general hospital costs are usually not too big, then the patient can afford for the costs—N1

Most of the patients are BPJS members, so money is not the problem for them, because everything is free?—N1

### 3.3. Acceptance of Health Workers

In terms of therapy selection, the data showed the diverse behavior between doctors and pharmacists. One participant revealed that there was a communication hierarchy which made the pharmacist’s advice difficult to accept. Pharmacist intervention is expected with the expectation that the service can run smoothly without any form of dispute.

In the emergency room, we also collided with hierarchy of the health professional. Sometimes the specialists ask for particular medicines. The pharmacist can discuss with the doctor for a change in medication if he runs out of medicine—P1.

Meanwhile, other participants could accept the pharmacist’s intervention as long as it was under the pharmacist’s confirmation, especially for narcotics with serious adverse drug reactions. Pharmacists have also confirmed that drugs that contain narcotics have side effects capable of endangering the life of patients.

I often get angry when someone changes medicine without confirmation if the drugs have some side effects that can endanger them—P2

In addition, other participants stated that the doctor was the decision maker and the person responsible for patient therapy. All decisions regarding therapy need to be properly communicated.

It’s just a matter of language, if communication is good, ok, if for example the communication is unclear, it doesn’t depend on the doctor—P4

In terms of medicine information services, one of the participants who happened to be a doctor stated that patients rarely asked them questions because they felt more comfortable asking someone who had similar knowledge and professional experience.

It was not a problem I did not bother asking them because I had a friend with similar professional knowledge—P3

### 3.4. Drug Information

The action of giving the drug information by pharmacists in emergency units is still limited. This is still often performed by other nurses and doctors and considered to be a waste of time, creating a greater workload. Pharmacists are still passive in dealing directly with patients. According to the participants, pharmacists are the most appropriate health workers and are competent with respect to drug information.

Actually, the activity of counseling, education and information to patients is suboptimal. The problem is that we ordered medication, which means that we are in the emergency unit and no pharmacists here. Clarifying prescriptions to patients will mean doubling the effort, so it will be better for me to give the medicine already explained by the pharmacy—P4.

So far communication is usually between pharmacists and nurses, and sometimes with patients which is most times rare—N2.

### 3.5. Expectations of Health Workers

#### 3.5.1. Drug Availability

Health workers expect pharmacists to do the drug procurement, thus avoiding shortages of medicine.

Other participants stated that drug supply was not the responsibility of other hospital personnel but rather that of the pharmacy. Other participant said that the drug supply is not only the responsibility of the pharmacist, but also other staff. Participants expected pharmacists to perform optimal drug planning, avoiding the unavailability of drugs. There are some techniques for the drug procurement, such as epidemiologic, consumption and the combination of epidemiologic-consumption. Pharmacists have competencies in this area, so that the other health professionals expect the best effort for the drug procurement.

#### 3.5.2. Drug Utilization Review

Overall, pharmacy services are also expected to be applied at the emergency department. According to participants, pharmacists do not only manage medicines and medical devices but also ensure the safety of patients. The pharmacy service should be in the emergency unit, especially when taking into account drug management, information, and patient safety. Collaboration with other health workers is expected to be focused on patient safety. It is also important that the pharmacist is given the responsibility of keeping track of a patient’s medical history. This will support inter professional communication between doctors and pharmacists. In giving therapeutic advice, one doctor stated that the pharmacist also needs to know the history of the drug so as to give the right advice that fits the needs of the individual.

The history of the treatment should be known to the pharmacist. So he can have an idea of the patients’ eligibility to take the drug. Yes, those are things that I think are necessary for adequate collaboration between doctors and pharmacists. Not all of the cases in the emergency unit are new—P2.

Pharmacist can remind us about the patient’s history, such as drug allergy—P4

Some doctors revealed that a detailed medical history can provide a good understanding of the patient’s condition and treatment. Not only is it beneficial, but it can also reduce workload and save service time.

We need it, maybe the pharmacists are more active in directing services to patients, and those who must be served. It is not right with us. Direct service means medicament delivery—P2.

#### 3.5.3. Drug Information and Education 

Drug information was expected to be delivered to the patients. This activity can decrease the workload of other health professionals, since currently, they also have to give information and education to the patients.

We need the pharmacist to give direct service to the patients, such as information and education—N2

Just give the drug information and education to the patients—P4

On the other hand, the nurses also expected the pharmacist to give the education about drug admixture. The nurses need more education about the incompatibility of drugs in the case of admixture.

Sometimes, we do not understand about how to admixture the drugs. In the past, we also did not learn about it—N2.

Training on how to admixture the drugs is given; however, it is necessary to increase the frequency and carry out routine checkups.

A few months ago there was but lately there is no such training—N4

The working environment and the large number of patients should be considered in order to reduce medication errors.

This means that many patients come into the emergency room, sometimes we also can’t blame human error because it can be tiring—P4

If the patients are few, it’s ok. But if there are many patients coming to ask for this, we get confused. Hassle—N3

## 4. Discussion

Medical information needs to be given in the emergency unit, covering a wide range of aspects including medicine selection, dosage and administration, medicine reactions that harm intravenous compatibility, drug interactions, and identification of unknown medicines. Pharmacists stationed at the emergency unit must ensure that appropriate access is provided at the primary, secondary, and tertiary levels to ensure the smooth and prompt respond to medical requests [5]. Simone et al. reported that almost 66% of nurses expected a protocol or informative brochure in the emergency room on how to admixture intravenous drugs [6]. Inadequate pharmacological knowledge and fatigue caused by high workload for nurses are some of the managerial and human factors that lead to medication error [7]. Another qualitative study revealed that the main factors that contribute in reducing drug safety problems include sustainable education for health workers, the development of a culture that encourages awareness for reporting medication errors, the use of technology, and the promotion and implementation of policies related to patient safety [8].

One of the impacts associated with the implementation of national health insurance is regulation and medical supply, which ultimately affect the availability of drugs. Lack of medication is mostly caused by high demand for certain types of needs such as cancer treatment and dialysis. The budgets for the purchase of medicines are routinely being siphoned to meet the shortage of cancer drugs [9]. According to Prabowo, one of the factors that influences the availability of medicine in the BPJS era is the inability of the pharmacist to procure adequate medicine [10]. A method to anticipate drug unavailability is substitution of drugs with other trade names that meet the criteria. More factors must be involved by the pharmacist in the drug procurement process, so that drug unavailability could be avoided.

A study about implementing a comprehensive pharmacist program in emergency unit, the department pharmacist program revealed an increase in cost efficiency, with 76.7% of respondents stating that the role of pharmacists could reduce the cost of healthcare [3,11]. According to a recent study, with pharmacy program in emergency unit, cost of efficiency reached 74.03%. This type of intervention can be said to be quite significant in terms of cost-saving. In that study, the health costs were not a concern for participants, and no significant cost savings were made by the pharmacists [12]. According to other research, the opportunity for cost savings in many countries is limited where the average cost for each patient per treatment represents only a small part of the total cost. Thus, it is difficult to justify the result [13].

In a study conducted by Keller et al. using a qualitative research approach, one participant stated hierarchy as one of the obstacles between doctors and nurses, and expressed relief that they could communicate outside the hospital with ease [14]. A similar study revealed that doctors might think that making prescriptions and ensuring the patient follows the instructions or orders is better than participating in decision-making. Differences in the educational level and knowledge between professions can impact members’ ability to exchange ideas and interpret patient health problems, thereby affecting the quality of treatment provided. This level of education and knowledge gap will hinder the process of effective communication [15]. A qualitative study conducted by Pun et al. revealed one of the problems of inter professional communication in the emergency unit as being the transfer of medical information to inadequate staff. Owing to the fact that patients often enter the emergency unit without medical records, a comprehensive understanding of the patient’s condition and treatment history should be made to support quality health services [16]. A drug utilization review should be conducted precisely in the emergency unit because of the inconsistency in the patient’s medical records. The pharmacist has the competence to perform drug reconciliation and to prevent medication errors during patient visits to the emergency unit [17,18].

Some pharmacy services can be developed at the emergency unit and this service involves creditors, regulators, and administrators of health care facilities [5]. For the clinical pharmacy practice in the hospital ward, normally one pharmacist must undertake prescription review, monitoring of treatment, giving of drug information, and education and counseling for 30 patients. For outpatient services, one pharmacist must perform prescription screening and drug administration, and provide information, education, and counseling for 50 patients [19]. According to this situation, ideally, one pharmacist must be placed in the emergency unit. 

For clinical practice, the pharmacy service in the emergency unit is still related to drug supply management. The national standard should be developed to increase the quality of the pharmacist services. The interprofessional relationship concept should be accepted and applied by all health professionals. This concept must be introduced in undergraduate education.

Health workers’ perceptions of the pharmacist’s role in the emergency unit include not only services but also the patients’ safety in the emergency unit. This perception brings up expectations about patient safety. On the other hand, the national health insurance system may influence perceptions and expectations. To the best of our knowledge, this study is the first qualitative study conducted in Indonesia on the topic of health workers’ perceptions of the pharmacist’s role in emergency units. Currently there is no national standard for the pharmacy services in emergency units. Our findings can be used as a consideration to develop the standard of the pharmacist’s role in the emergency unit. Physicians and nurses perceive that delivering the drug information is an important job of the pharmacist in the emergency unit. However, the pharmacist’s role can be limited by national health insurance due to the limited medications available in the emergency unit. During emergency situations, the physician and the nurse need medications appropriate to the patients’ conditions. The pharmacist is responsible for the availability of the medications.

This study has the limitation due to the use of only one method, which was the semi structured interview method. Thus, we cannot generalize the results. The results of study were more focused on drug supply management and drug information. In these cases, the small number of participants can support the validity of the study findings. Furthermore, data analysis was only carried out by the researcher to clarify the efficiency of the data obtained. 

## 5. Conclusions

Various perceptions are conveyed by participants regarding the pharmacy service in emergency units. The existence of a pharmacist may increase the efficiency of service time and prevent human error. The national health insurance service is in charge of the role played by health workers and pharmacists. Communication, information, and adequate medical education are still insufficient in emergency units. Participants expect that pharmacists and policy makers make adequate provisions for drugs in the emergency units. Pharmacists need to enhance the quality of practice in accordance with their competence, especially in tracking the patient’s drug history and in terms of drug education for both patients and health workers. These expectations are intended reduce the workload of participants, save time, and improve the quality of communication among medical personnel.

## Figures and Tables

**Table 1:** Participants’ Characteritics.

Characteristics	*N*	%
**Age**		
<30	2	25
30–40	3	37.5
40–50	3	37.5
**Gender**		
Male	4	50
Female	4	50
**Profession**		
General physician	4	50
Nurse	4	50
**Working (Mean 7.4 years)**		
<10 years	4	50
>10 years	4	50

**Table 2:** Theme classifications and participant quotes.

Major Themes	Examples
Positive impact of pharmacists in service	*When a pharmacist comes across a problem associated with wrong dosage, he will review it. Luckily for me, mine was very careful, we could not just take medicine without his assistance—N1.*
Effect of National Insurance (Badan Penyelenggara Jaminan Sosial:BPJS)	*We are more flexible with BPJS patients because certainty is guaranteed, but in the case of accident victims, we sometimes ask them to pay upfront, most times they ask us to treat them with the guarantee of paying up later. However, sometimes they do not redeem these bills. So it’s wrong too—P4*
Acceptance of health workers	*It’s just a matter of language, if communication is good, ok, if for example the communication is insistent, it doesn’t depend on the doctor—P4*
Drug information	*Same is applicable with medicine. All who understand the medicine are pharmacists. It never goes down to the patient—P2.*
Expectations of health workers	*If we had the slightest idea that this drug would be finishing soonest, we would have made adequate provision for it. Since it hasn’t been replaced till now, we are assuming it must have run out of production—N2.* *It is expected that more drugs are purchased, especially if there is an emergency—N4.* *It is not only about the pharmacist. Pharmacists are all good, and they ensure the availability of medicine in hospitals—P1.* *Pharmacists must understand that the drugs are fast moving, so they can do the planning of the procurement better—N2.* *Pharmacists have to increase their effort in planning the procurement of drug in the emergency unit—P4.*
